# Supplementary material for: The Impact of Complement Genes on the Risk of Late-Onset Alzheimer’s Disease
Source: Genes (Basel). 2021 Mar 20;12(3):443. doi: 10.3390/genes12030443 (PMC8003605; doi:10.3390/genes12030443)
Supplement: Supplementary file 1 [file genes-12-00443-s001.pdf]

Supplementary file 1: List of GERAD authors and affiliations

Genetic and Environmental Risk in AD/Defining Genetic, Polygenic and Environmental Risk for Alzheimer's Disease Consortium (GERAD/PERADES) Consortium

Rebecca Sims[1], Maria Vronskaya[2], Aura Frizatti[2], Taniesha Morgan[2], Nicola Denning[1], Thomas D Cushion[1,2], Lesley Jones[2], Rachel Marshall[2], Alun Meggy[1], Georgina Menzies[1], Ganna Leonenko[1], Detelina Grozeva[2], Michael C O'Donovan[2], Michael J Owen[2], Valentina Escott-Price[1,2], Peter A Holmans[2], Julie Williams[1,2], Magda Tsolaki[3], David Craig[4], Despoina Avramidou[5], Antonia Germanou[5], Maria Koutroumani[5], Olymbia Gkatzima[5], Harald Hampel[6,7,8], Patrick G Kehoe[9], Seth Love[9], David C Rubinsztein[10], Lutz Frölich[11], Bernadette McGuiness[12], Janet A Johnston[12], Peter Passmore[12], Dmitriy Drichel[13], Nick C Fox[14], Martin Rossor[14], Jonathan M Schott[14], Jason D Warren[14], Jose Bras[15,16,17], Rita Guerreiro[15,16,17], Amit Kawalia[18], Joseph T Hughes[19], Yogen Patel[19], Michelle K Lupton[19,20], Petra Proitsi[19,20], John Powell[19,20], John S K Kauwe[21], Michelangelo Mancuso[22], Ubaldo Bonuccelli[22], John Hardy[15,23], James Uphill[24], Elizabeth Fisher[24], Carlo Masullo[25], Hilkka Soininen[26], Gina Bisceglio[27], Li Ma[27], Dennis W Dickson[27], Neill R Graff-Radford[27], Minerva M Carrasquillo[27], Steven G. Younkin[27,28], Sandro Sorbi[29,30], Makrina Daniilidou[31], Angela Hodges[32], Daniela Galimberti[33,34], Elio Scarpini[33,34], Dan Rujescu[35], Martin Scherer[36], Alfredo Ramirez[37,38,39,40], Markus Leber[41], Sabrina Pichler[42], Manuel Mayhaus[42], Wei Gu[42], Matthias Riemenschneider[42], Jens Wiltfang[43,44,45], Reinhard Heun[46], Heike Kölsch[46], Johannes Kornhuber[47], Isabella Heuser[48], Annette M Hartmann[49], Ina Giegling[49], Michael Hüll[50], Simon Lovestone[51], Carlos Cruchaga[52,53], John Morris[52,53], Kevin Mayo[54], Thomas Feulner[55], Rebecca Sussams[56], Clive Holmes[56], David Mann[57], Stuart Pickering-Brown[57], Nigel M Hooper[57], Andrew McQuillin[58], Gill Livingston[58], Nicholas J Bass[58], Elisa Toppi[59], Paola Bossù[59], Giancarlo Russo[60], Wolfgang Maier[46,61], Frank Jessen[46,61], H-Erick Wichmann[62,63,64], Kevin Morgan[65], Alison M Goate[66,67], Bruno Vellas[68], Emma Vardy[69], Susanne Moebus[70], Karl-Heinz Jöckel[70], Martin Dichgans[71,72], Norman Klopp[73], James Turton[74], Jenny Lord[74], Kristelle Brown[74], Christopher Medway[74], Markus M Nöthen[75], Per Hoffmann[75,76], Antonio Daniele[77], Anthony Bayer[78], John Gallacher[78], Hendrik van den Bussche[79], Carol Brayne[80], Steffi Riedel-Heller[81], John F Powell[82], Ammar Al-Chalabi[82], Christopher E Shaw[83], Iwona Kloszewska[84], Aoibhinn Lynch[85], Brain Lawlor[85], Michael Gill[85], Eliecer Coto[86], Victoria Alvarez[86], Andrew B Singleton[87], John Colinge[88], Simon Mead[88], Natalie Ryan[88], Benedetta Nacmias[89,90], Sara Ortega-Cubero[91,92,93], Jacob Shofany[94], Nerisa Banaj[94], Valentina Ciullo[94], Eleonora Sacchinelli[94], Gianfranco Spalletta[94], Robert Clarke[95], A David Smith[96], Donald Warden[96], Yoav Ben-Schlomo[97], Chiara Cupidi[98], Raffaele Giovanni Maletta[98], Amalia Cecilia Bruni[98], Maura Gallo[98], Denise Harold[99], Roberta Cecchetti[100], Patrizia Mecocci[100], Pau Pastor[101], Virginia Boccardi[100], Monica Diez-Fairen[101], Nick Warner[102], Gordon Wilcock[103], Panagiotis Deloukas[104], Rhian Gwilliam[104], Chris Corcoran[105], JoAnn Tschanz[105], Ron Munger[105], Eloy Rodriguez-Rodriguez[106], Pascual Sanchez-Juan[106].

1 UKDRI at Cardiff University, Cardiff, UK

2 Division of Psychological Medicine and Clinial Neurosciences, MRC Centre for Neuropsychiatric Genetics and Genomics, Cardiff University, UK

3 3rd Department of Neurology, Medical School, Aristotle University of Thessaloniki, Thessaloniki, Greece

4 Ageing Group, Centre for Public Health, School of Medicine, Dentistry and Biomedical Sciences, Queen's University Belfast, UK

5 Aristotle University of Thessaloniki, Despere 3, Thessaloniki, 54621, Greece
6 AXA Research Fund & UPMC Chair, Paris, France
7 Sorbonne Universités, Université Pierre et Marie Curie, Paris
8 Institut de la Mémoire et de la Maladie d'Alzheimer (IM2A) & Institut du Cerveau et de la Moelle épinière (ICM), Département de Neurologie, Hôpital de la Pitié-Salpêtrière, Paris, France
9 Bristol Medical School, University of Bristol, Southmead Hospital, Bristol, UK
10 Cambridge Institute for Medical Research and UK Dementia Research Institute, University of Cambridge, Cambridge, UK
11 Central Institute of Mental Health, Medical Faculty Mannheim, University of Heidelberg, Germany
12 Centre for Public Health, School of Medicine, Dentistry and Biomedical Sciences, Queens University, Belfast, UK
13 Cologne Center for Genomics, University of Cologne, Cologne, Germany
14 Dementia Research Centre, Department of Neurodegenerative Disease, UCL Institute of Neurology, London, UK
15 UKDRI at UCL, London, UK
16 Department of Molecular Neuroscience, UCL, Institute of Neurology, London, UK
17 Department of Medical Sciences, Institute of Biomedicine iBiMED, University of Aveiro, 3810-193 Aveiro, Portugal
18 Department for Neurodegenerative Diseases and Geriatric Psychiatry, University Hospital Bonn, Bonn, Germany
19 Department of Basic and Clinical Neuroscience, Institute of Psychiatry, Psychology and Neuroscience, Kings College London, London UK
20 Genetic Epidemiology, QIMR Berghofer Medical Research Institute, Herston, Queensland, Australia
21 Department of Biology, Brigham Young University, Provo, Utah, USA
22 Department of Experimental and Clinical Medicine, Neurological Institute, University of Pisa, Italy
23 Department of Molecular Neuroscience, UCL, Institute of Neurology, London, UK
24 Department of Neurodegenerative Disease, MRC Prion Unit, UCL Institute of Neurology, London, UK
25 Department of Neurology, Catholic University of Rome, Rome, Italy
26 Department of Neurology, University of Eastern Finland and Kuopio University Hospital, Kuopio, Finland
27 Department of Neuroscience, Mayo Clinic, Jacksonville Florida 32224
28 Department of Neurology, Mayo Clinic, Jacksonville, Florida, USA
29 Department of Neuroscience, Psychology, Drug Research and Child Health, University of Florence, Italy Viale Pieraccini 6, 50139 Florence, Italy
30 IRCCS Don Gnocchi , Florence, Italy
31 Department of Neuroscience, Uppsala University, Uppsala, Sweden
32 Department of Old Age Psychiatry, Institute of Psychiatry, Psychology and Neuroscience, Kings College London, London UK
33

Dept. of Biomedical, Surgical and Dental Sciences, University of Milan, Milan, IT
34 Fondazione IRCCS Ca' Granda, Ospedale Policlinico, Milan, IT
35 Department of Psychiatry, Martin-Luther-University Halle-Wittenberg, Halle, Germany
36 Department of Primary Medical Care, University Medical Centre Hamburg-Eppendorf, 20246 Hamburg, Germany
37Division of Neurogenetics and Molecular Psychiatry, Department of Psychiatry and Psychotherapy, University of Cologne, Medical Faculty, 50937 Cologne, Germany.
38 Department of Neurodegenerative diseases and Geriatric Psychiatry, University of Bonn, 53127 Bonn, Germany.
39 German Center for Neurodegenerative Diseases (DZNE), 53127 Bonn, Germany.
40 Department of Psychiatry and Glenn Biggs Institute for Alzheimer's and Neurodegenerative Diseases, San Antonio, TX, USA
41 Department of Psychiatry and Psychotherapy, University Hospital Cologne, Cologne, Germany
42 Department of Psychiatry and Psychotherapy, University Hospital, Saarland, Germany
43 Department of Psychiatry and Psychotherapy, University Medical Center (UMG), Georg-August-University, von-Siebold-Strasse 5, 37075 Göttingen
44 German Center for Neurodegenerative Diseases (DZNE), Göttingen, Germany
45 Institute for Research in Biomedicine (iBiMED), Medical Sciences Department, University of Aveiro, Aveiro, Portugal
46 Department of Psychiatry and Psychotherapy, University of Bonn, 53127, Bonn, Germany
47 Department of Psychiatry and Psychotherapy, University of Erlangen-Nuremberg, Germany
48 Department of Psychiatry, Charité Berlin, Germany
49 Department of Psychiatry, Martin-Luther-University Halle-Wittenberg, Halle, Germany
50 Department of Psychiatry, University of Freiburg, Freiburg, Germany (M.H.)
51 Department of Psychiatry, University of Oxford, Oxford, UK
52 Department of Psychiatry, Washington University School of Medicine, St. Louis Missouri, USA
53 Hope Center Program on Protein Aggregation and Neurodegeneration, Washington University School of Medicine, St. Louis, Missouri, USA
54 Departments of Psychiatry, Neurology and Genetics, Washington University School of Medicine, St Louis, MO 63110, US.
55 Dept. Of Psychaitry, University Hospital, Saarland
56 Division of Clinical Neurosciences, School of Medicine, University of Southampton, Southampton, UK
57 Division of Neuroscience and Experimental Psychology, School of Biological Sciences, Faculty of Biology, Medicine and Health, University of Manchester, Manchester Academic Health Science Centre, Manchester M13 9PT, UK
58 Division of Psychiatry, University College London, UK
59 Experimental Neuropsychobiology Laboratory, IRCCS Santa Lucia Foundation, Department of Clinical and Behavioral Neurology, Rome, Italy
60 Functional Genomics Center Zurich, ETH/University of Zurich
61 German Center for Neurodegenerative Diseases (DZNE), 53127 Bonn, Germany
62 Helmholtz Center Munich, Institute of Epidemiology, Neuherberg
63 Ludwig-Maximilians University Chair of Epidemiology, Munich, Germany

64 Joint Biobank Munich and KORA Biobank
65 Human Genetics, School of Life Sciences, Life Sciences Building A27, University Park, University of Nottingham, Nottingham, NG7 2RD, United Kingdom
66 Icahn School of Medicine at Mount Sinai, New York, NY, USA
67 Hope Center Program on Protein Aggregation and Neurodegeneration, Washington University School of Medicine, St Louis, Missouri, USA
68 INSERM U 558, University of Toulouse, Toulouse, France
69 Institute for Ageing and Health, Newcastle University, Biomedical Research Building, Campus for Ageing and Vitality, Newcastle upon Tyne
70 Institute for Medical Informatics, Biometry and Epidemiology, University Hospital of Essen, University Duisburg-Essen, Hufelandstr, 55, D-45147 Essen, Germany
71 Institute for Stroke and Dementia Research, Klinikum der Universität München, Munich, Germany
72 German Center for Neurodegenerative Diseases (DZNE, Munich), Munich, 80336, Germany
73 Institute of Epidemiology, Helmholtz Zentrum München, German Research Center for Environmental Health, Neuherberg, Germany
74 Institute of Genetics, Queens Medical Centre, University of Nottingham, Nottingham, UK
75 Institute of Human Genetics, Department of Genomics, Life and Brain Center, University of Bonn, Bonn, Germany
76 Division of Medical Genetics, University Hospital and Department of Biomedicine, University of Basel, CH-4058, Basel, Switzerland
77 Institute of Neurology, Catholic University of Sacred Hearth, Rome, Italy
78 Institute of Primary Care and Public Health, Cardiff University, Neuadd Meirionnydd, University Hospital of Wales, Heath Park, Cardiff UK
79 Institute of Primary Medical Care, University Medical Center Hamburg-Eppendorf, Germany
80 Institute of Public Health, University of Cambridge, Cambridge, UK
81 Institute of Social Medicine, Occupational Health and Public Health, University of Leipzig, 04103 Leipzig, Germany
82 King's College London, Institute of Psychiatry, Department of Neuroscience, De Crespigny Park, Denmark Hill, London SE5
83 UKDRI at King’s College London, London, UK
84 Medical University of Lodz, Lodz, Poland
85 Mercer's Institute for Research on Ageing, St James' Hospital, Dublin 8
86 Molecular Genetics Lab-Hospital Univ Central Asturias, Oviedo, Spain, 33011-Oviedo-Spain
87 Molecular Genetics Section, Laboratory of Neurogenetics, National Institute on Aging, National Institutes of Health, Bethesda, MD 20892, USA
88 MRC Prion Unit at UCL, Institute of Prion Diseases, London
89 NEUROFARBA (Department of Neuroscience, Psychology, Drug Research and Child Health), University of Florence, Florence, Italy
90 Centro di Ricerca, Trasferimento e Alta Formazione DENOTHE, University of Florence, Florence, Italy
91 Neurogenetics Laboratory, Division of Neurosciences, Centre for Applied Medical Research, University of Navarra School of Medicine, Pamplona, Spain

92 CIBERNED, Centro de Investigación Biomédica en Red de Enfermedades Neurodegenerativas, Instituto de Salud Carlos III, Madrid, Spain
93 Department of Neurology, Complejo Asistencial Universitario de Palencia, Spain
94 Neuropsychiatry Laboratory, IRCCS Santa Lucia Foundation, Department of Clinical and Behavioral Neurology, Rome, Italy
95 Oxford Healthy Aging Project (OHAP), Clinical Trial Service Unit, University of Oxford, Oxford, UK
96 Oxford Project to Investigate Memory and Ageing (OPTIMA), University of Oxford, Level 4, John Radcliffe Hospital, Oxford OX3 9DU, UK
97 Population Health Sciences, Bristol Medical School, University of Bristol
98 Regional Neurogenetic Centre (CRN), ASP Catanzaro, Lamezia Terme, Italy
99 School of Biotechnology, Dublin City University, Dublin 9, Ireland
100 Section of Gerontology and Geriatrics, Department of Medicine, University of Perugia, Perugia, Italy
101 Memory Disorders Unit, Department of Neurology, University Hospital Mutua de Terrassa, Terrassa, Barcelona, Spain.
102 Somerset Partnership NHS Trust, Somerset, UK
103 The Oxford Project To Investigate Memory and Ageing, Nuffield Department of Clinical Neurosciences, University of Oxford, West Wing, John Radcliffe Hospital, Oxford. OX3 9DJ.
104 The Wellcome Trust Sanger Institute, Wellcome Trust Genome Campus, Hinxton, Cambridge, UK.
105 Utah State University, Logan, Utah, USA
106 Neurology Service and Centro de Investigacion Biomedica en Red sobre Enfermedades Neurodegenerativas (CIBERNED), 'Marques de Valdecilla' University Hospital, University of Cantabria, Institute for Research, 'Marques de Valdecilla' (IDIVAL), Santander, Spain
